# In Silico Prediction of Plasma Concentrations of Fluconazole Capsules with Different Dissolution Profiles and Bioequivalence Study Using Population Simulation

**DOI:** 10.3390/pharmaceutics11050215

**Published:** 2019-05-05

**Authors:** Marcelo Dutra Duque, Daniela Amaral Silva, Michele Georges Issa, Valentina Porta, Raimar Löbenberg, Humberto Gomes Ferraz

**Affiliations:** 1 Department of Pharmacy, Faculty of Pharmaceutical Sciences, Universidade de São Paulo—USP, Av. Prof. Lineu Prestes, 580, São Paulo—SP 05508-000, Brazil; amaralsi@ualberta.ca (D.A.S.); michelegeorges@usp.br (M.G.I.); vporta@usp.br (V.P.); sferraz@usp.br (H.G.F.); 2 Department of Pharmaceutical Sciences, Institute of Environmental, Chemical and Pharmaceutical Sciences, Universidade Federal de São Paulo—UNIFESP, Rua São Nicolau, 210, Centro, Diadema—SP 09913-030, Brazil; 3 Faculty of Pharmacy & Pharmaceutical Sciences, Katz Group-Rexall Centre for Pharmacy & Health Research, University of Alberta, 11361 – 87 Avenue, Edmonton, AB T6G 2E1, Canada; raimar@ualberta.ca

**Keywords:** fluconazole, biowaiver, dissolution, Biopharmaceutics Classification System (BCS), bioequivalence, GastroPlus^™^

## Abstract

A biowaiver is accepted by the Brazilian Health Surveillance Agency (ANVISA) for immediate-release solid oral products containing Biopharmaceutics Classification System (BCS) class I drugs showing rapid drug dissolution. This study aimed to simulate plasma concentrations of fluconazole capsules with different dissolution profiles and run population simulation to evaluate their bioequivalence. The dissolution profiles of two batches of the reference product Zoltec^®^ 150 mg capsules, A1 and A2, and two batches of other products (B1 and B2; C1 and C2), as well as plasma concentration–time data of the reference product from the literature, were used for the simulations. Although products C1 and C2 had drug dissolutions < 85% in 30 min at 0.1 M HCl, simulation results demonstrated that these products would show the same in vivo performance as products A1, A2, B1, and B2. Population simulation results of the ln-transformed 90% confidence interval for the ratio of *C*_max_ and AUC_0–t_ values for all products were within the 80–125% interval, showing to be bioequivalent. Thus, even though the in vitro dissolution behavior of products C1 and C2 was not equivalent to a rapid dissolution profile, the computer simulations proved to be an important tool to show the possibility of bioequivalence for these products.

## 1. Introduction

Bioavailability (BA) is the rate and extent of absorption of an active pharmaceutical ingredient from a dosage form when it becomes available at the site of action. Drug products that are pharmaceutical equivalents are considered bioequivalent and, therefore, interchangeable when BA is not statistically different between the two products after administration at the same dose and under similar experimental conditions in a bioequivalence (BE) study. For purposes of establishing BE, a test product must be compared to a reference product [1,2,3]. 

For highly soluble and highly permeable drugs, categorized as class I according to the Biopharmaceutics Classification System (BCS), the waiver of BE studies can be considered in immediate-release (IR) products [4]. In this case, the rate and extent of absorption are not dependent on drug dissolution but rather solely on gastric emptying [5]. In IR products containing BCS class I drugs with rapid (≥85% in 30 min) or very rapid (≥85% in 15 min) in vivo dissolution in relation to gastric emptying, BA is independent of drug dissolution or gastrointestinal transit time [4].

The Brazilian Health Surveillance Agency (ANVISA) guidance for biowaiver [6] allows the waiver of bioequivalence studies for BCS class I drugs that are described in a Normative Instruction [7], which was updated later [8]. For this purpose, the applicant should provide data demonstrating rapid drug dissolution from the IR dosage form, i.e., at least 85% of the drug dissolved within 30 min under all conditions tested for both test and reference products [6]. The dissolution test conditions accepted by ANVISA are suggested in the Pharmaceutical Equivalence and Comparative Studies of Dissolution Profiles guidance [9]: apparatus 1 at 100 rpm or apparatus 2 at 50 rpm; dissolution media pH 1.2 HCl or simulated gastric fluid without enzyme, pH 4.5 and pH 6.8 (or simulated intestinal fluid); 900 mL and temperature 37 ± 1 °C.

Fluconazole is a triazole antifungal agent indicated for superficial and systemic infections, and is available for oral administration in capsules containing 50–150 mg of the drug; it is generally well absorbed, showing a BA of about 90% [10]. Due to its clinical and biopharmaceutical characteristics, fluconazole is a candidate for BCS class I biowaiver [11,12]. This drug is described in the Brazilian Health Surveillance Agency’s Normative Instruction list of drug candidates for the waiver of bioequivalence studies according to BCS [8]. 

Currently, the prediction of intestinal absorption of drugs based on computer simulations using advanced compartmental absorption and transit (ACAT) and physiologically-based pharmacokinetic (PBPK) models is a reality [13,14,15,16,17,18,19,20,21]. Computer software can be used to predict oral absorption of drugs from IR products containing different dissolution profiles, helping formulation scientists to decide on the best dissolution test conditions or formulation, gaining time, and reducing costs in drug development [17]. Computer simulations have been demonstrated to also be important for biowaiver extension for BCS class II [14,22,23] and class III drugs [24]. 

The dissolution criterion for the biowaiver (rapid or very rapid dissolution) of IR products containing 32 BCS class I drugs was evaluated by [25], which showed that very rapid dissolution is not necessary to guarantee BE according to BCS. However, considering the importance of appropriate dissolution tests and the possibility of requesting a waiver of BE studies when in vitro dissolution data are available for BCS class I drugs [3,6], the objective of this article was to use computer simulations to predict the plasma concentrations of fluconazole capsules with different dissolution profiles using the software GastroPlus^™^, comparing products with rapid or very rapid drug dissolution to products that do not meet this criterion, and evaluating whether they could be bioequivalent using simulated population studies. 

## 2. Methods

### 2.1. Plasma Concentration Simulations

The software GastroPlus^™^ version 9.0 (Simulations Plus Inc., Lancaster, CA, USA) was used to predict the oral absorption of fluconazole 150 mg capsules from different manufacturers compared to the reference product in Brazil, Zoltec^®^ 150 mg capsules (Laboratórios Pfizer Ltda, Guarulhos, Brazil).

For such, a fluconazole database was created in GastroPlus^™^. Input data consisted of values taken from the literature, including but not limited to drug solubility, pKa, and the logarithm of the partition coefficient (Log P), as well as other parameters obtained using the ADMET Predictor^™^ (Absorption, Distribution, Metabolism, Elimination and Toxicity Predictor) (Simulations Plus, Lancaster, CA, USA) module in GastroPlus^™^ (Table 1).

Plasma concentrations of the reference product (Zoltec^®^ 150 mg capsules) previously reported by [28] were extracted from the original figure using Web Plot Digitizer [30] and used in the PKPlus^™^ module in GastroPlus^™^ to build a compartmental pharmacokinetic (PK) model. PKPlus^™^ is an optional module in GastroPlus^™^ that uses intravenous or oral plasma concentration–time data to calculate the most appropriate modeling (one-, two-, or three-compartmental models) and generate PK parameters for simulations [31].

In the GastroPlus^™^ fluconazole database, records were created for two batches of three different products, namely A1, A2, B1, B2, C1, and C2, as previously described by [29]; A1 and A2 were batches #1 and #2, respectively, of the reference product Zoltec^®^ 150 mg capsules, whereas B1, B2, C1, and C2 were batches #1 and #2 of two different products found in the Brazilian market. The dissolution tests were performed using the United States Pharmacopeia (USP) apparatus 1 (basket) with 900 mL of 0.1 M HCl at 37 ± 0.5 °C and 100 rpm for 30 min and yielded results (*n* = 6 for each product) [29] that were used in the software to simulate the plasma concentration for each product (A1, A2, B1, B2, C1, and C2). 

The simulations were run in GastroPlus^™^ in order to obtain the predicted values of the plasma concentration. Concentration curves were compared to that of the reference product (Ref) with respect to regression parameters generated by the software: coefficient of determination (R^2^), sum of square error (SSE), root mean square error (RMSE), and mean absolute error (MAE).

The pharmacokinetic parameters %F (% bioavailable), which is the percent of drug that reached the systemic circulation, *T*_max_, *C*_max_, and AUC (Area Under the Curve) were generated by GastroPlus^™^. 

### 2.2. Population Simulations

Population Simulator is one of the simulation modes present in GastroPlus^™^. This mode allows the user to run simulated clinical trials for virtual subjects, combining physiology and pharmacokinetic variability within populations and considering formulation variables used as input data in the software. In this mode, Monte Carlo simulations are used to randomly generate each subject to have a unique set of parameters (gastrointestinal transit time and pH; pharmacokinetic parameters; plasma protein binding; small intestine, stomach, caecum and colon dimensions; and hepatic blood flow rate). Variations in each parameter are randomly generated for each simulated virtual subject [31].

In this way, population simulations were run in GastroPlus^™^ for each product (A1, A2, B1, B2, C1, and C2) considering in the Population Simulator mode the number of output data points to be saved for each virtual subject as 25. The number of virtual subjects chosen for the simulations was 28. This was the same number of volunteers enrolled in the bioequivalence study described by reference #28. Bioequivalence between each product and the reference Zoltec^®^ 150 mg capsules [28] from population simulation results were presented as 90% confidence interval (CI) for the ratio of *C*_max_ and AUC_0–t_ using ln-transformed data. GastroPlus^™^ automatically set in green the values of 90% CI for *C*_max_ and AUC_0–t_ that are within the 80–125% interval, according to regulatory agencies [32,33]. The end time used for the simulations was 96 h and the average AUC_0–t_/AUC_0–inf_ ratio was calculated.

## 3. Results and Discussion

After adding plasma concentrations of the reference product, Zoltec^®^ 150 mg capsules, in the software GastroPlus^™^ and selecting compartmental PK modeling, the PKPlus^™^ module calculated the most appropriate compartmental model (one, two, or three compartments) considering the administration of fluconazole 150 mg as IR capsules to subjects with an average weight of 61 kg under fasting conditions. Compartmental models were compared by evaluating R^2^ and the Akaike Information Criterion (AIC). As shown in Table 2, the two-compartmental model presented the best fit due to the highest R^2^ and lowest AIC value. Table 3 presents the PK parameters predicted using the selected model.

Mean (± standard deviation, SD) clearance (CL) and central compartment volume (Vc) values for fluconazole in healthy subjects are reported in the literature as 1.272 ± 0.219 L/h and 46.3 ± 7.9 L (38.4 to 54.2 L), respectively [34], whereas T1/2 of fluconazole is about 30 h [10]. Thus, the CL value calculated using PKPlus^™^ is in accordance with the literature, and the calculated Vc is near to the lowest (38.4 L) value reported by [34].

**Figure 1:**
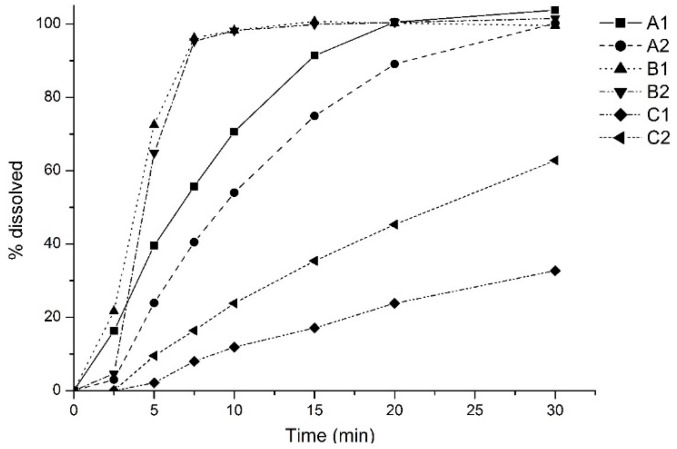
Dissolution profiles of products A1, A2, B1, B2, C1, and C2, obtained in USP Apparatus 1 (basket) with 900 mL of 0.1 M HCl at 37 ± 0.5 °C and 100 rpm for 30 min (adapted from [29], with permission from *Brazilian Journal of Pharmaceutical Sciences*, 2019).

Figure 1 shows the percent of fluconazole dissolved over time during dissolution tests of products A1, A2, B1, B2, C1, and C2 [29].

Products A1, A2, B1, B2, C1, and C2 had 91.4%, 74.9%, 100.7%, 99.9%, 17.1%, and 35.4% of drug dissolved in 15 min, respectively. Although product A2 have shown less than 85% of the drug dissolved in 15 min, only products C1 and C2 had values that did not reach 85% of drug dissolved at the end of the test (Figure 1). Considering these findings, products C1 and C2 are not expected to be bioequivalent to the reference product.

Fluconazole is a high solubility and high permeability (BCS class I) drug, which is not expected to present problems in dissolution tests. However, Charoo et al. [35] described the influence of the polymorphism of fluconazole on dissolution. This drug exhibits the polymorphic forms I, II, III, and monohydrate. The solubility values of forms I and monohydrate were reported as 4.96 and 4.21 mg/mL, respectively, and form II as 6.59 mg/mL in water at 25 °C [36,37]. Low intrinsic dissolution rate was also reported for the form I [38,39]; the tendency of form II to convert into forms I and monohydrate in the presence of high humidity values have also been reported [40]. It is possible that products C1 and C2 contain polymorphic form I or monohydrate due to the lower in vitro dissolution rate in comparison to the other products, as shown in Figure 1. 

**Figure 2:**
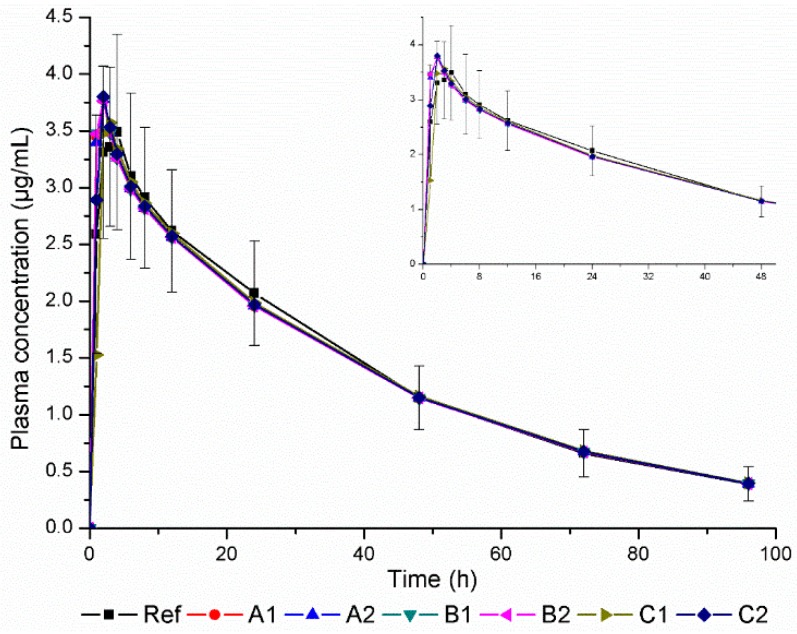
Plasma concentration-time curve of reference product (Ref) based on experimental values and of products A1, A2, B1, B2, C1, and C2 created using simulated values given by GastroPlus^™^; error bars represent the standard deviation for the reference plot. A zoom in of the time period 0–48 h is highlighted for better visualization.

GastroPlus^™^ was used to simulate plasma concentration–time curves for all the products, including C1 and C2, and the resulting profiles were then compared to the reference product (Ref) curve plotted with experimental values (Figure 2) in order to evaluate in vivo performance.

According to the predicted plasma concentration–time curves (Figure 2), all products would show in vivo performance equivalent to that of the reference product. 

Statistical parameters (*R*^2^, SSE, RMSE e MAE) generated by the software for each predicted profile in comparison to the plasma concentration–time curve of the reference product [28] are shown in Table 4.

For all predicted profiles, low values were found for the prediction error parameters SSE, RMSE, and MAE (Table 4), indicating the viability of using GastroPlus^™^ to predict plasma concentrations of fluconazole from input data. High correlation (*R*^2^) was demonstrated between the predicted plasma concentration–time curves of all products and the experimentally-determined reference curve.

The percent of drug dissolved less than 85% at 30 min for products C1 and C2 did not affect the predicted in vivo performance, as observed in the predicted plasma concentration–time curves (Figure 2) and statistical parameters (Table 4).

**Figure 3:**
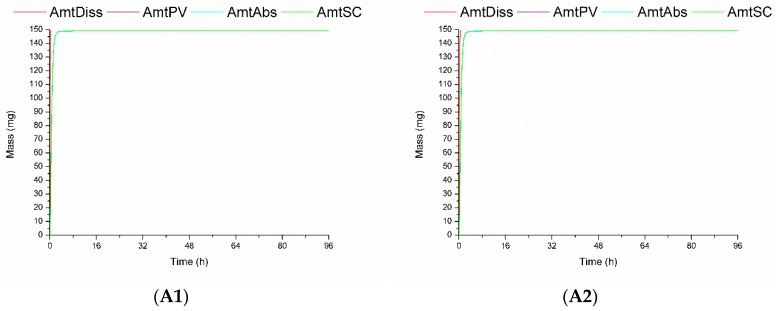

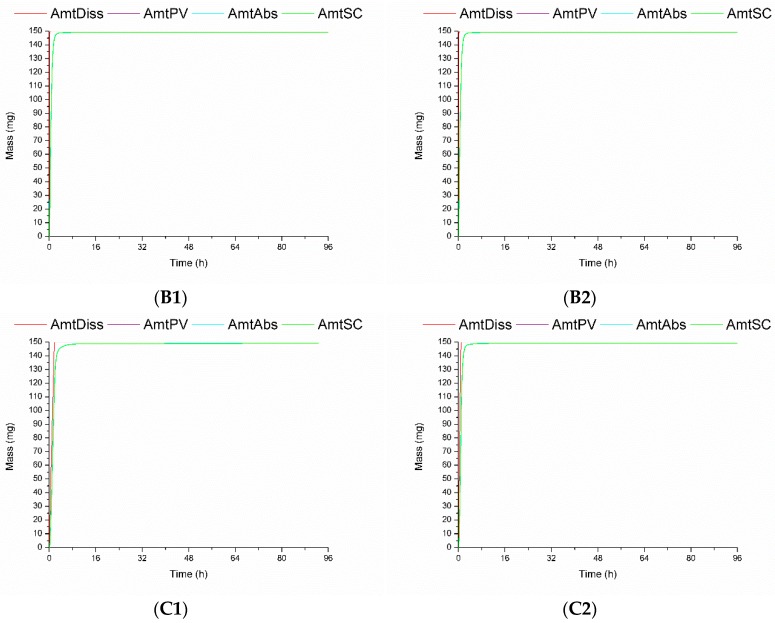
Amount of fluconazole dissolved in vivo (AmtDiss), amount absorbed to the portal vein (AmtPV), total amount absorbed (AmtAbs), and the amount in the systemic circulation (AmtSC) for products (**A1**–**C2**) calculated by GastroPlus^™^. Simulation time displayed is 8 h instead of the total simulation time (96 h) to provide better visualization.

GastroPlus^™^ also calculates the amount of drug dissolved in vivo (AmtDiss), absorbed to the portal vein (AmtPV), total amount absorbed (AmtAbs), and the amount in the systemic circulation (AmtSC). These data are shown in Figure 3.

It can be observed in Figure 3 that products A1, A2, B1, and B2 showed similar predicted in vivo dissolution behavior with a fast amount absorbed by enterocytes, then into the portal vein, and in the systemic circulation. The slow in vitro dissolution of products C1 and C2 (Figure 1) could be attributed to the presence of the low soluble polymorphic forms I and monohydrate. Despite a small displacement of the curves (Figure 3) corresponding to the predicted in vivo dissolution (AmDiss) of products C1 and C2 was observed, it did not affect the predicted fluconazole amount absorbed (AmtAbs) and the predicted amount in the systemic circulation (AmtSC). It is expected that for fluconazole, as for BCS class I drugs, dissolution is not the limiting step for absorption. 

The *C*_max_ and AUC predicted values for products A1, A2, B1, B2, C1, and C2 and the experimental ones for the reference product (Ref) are shown in Table 5. 

As stated in the Methods section, in vivo data of the reference product (Ref) was extracted from a figure containing the plasma concentration–time curve of Zoltec^®^ 150 mg capsules described by the reference #28, using WebPlotDigitizer [30]. These in vivo values were used as input data of Ref in GastroPlus^™^ and the pharmacokinetic parameters (F%, *T*_max_, *C*_max_, and AUC_0–t_) presented as Obs (Table 5) were calculated by the software. These PK parameters are in accordance with those reported by reference #28 (*T*_max_ = 2.96 h (1.96 h−3.96 h), *C*_max_ = 3.64 µg/mL (2.85 µg/mL–4.43 µg/mL), and AUC_0–t_ = 135.72 µg h/mL (106.20 µg h/mL–165.24 µg h/mL)). Even with the small difference found in the value of *T*_max_, the calculations made using GastroPlus^™^ were reasonable.

ANVISA recommendations for the waiver of BE studies according to BCS for class I drugs state that IR solid oral products must have rapid drug dissolution (≥85% in 30 min) in 0.1 M HCl, pH 4.5 and pH 6.8 [6]. In this study, even though the dissolution tests were carried out only in hydrochloric acid as the dissolution medium [29], products C1 and C2 that did not meet the requirements of the dissolution rate still showed in vivo performance equivalent to the reference product. Al-Tabakha et al. [41] evaluated different products containing amoxicillin trihydrate and potassium clavulanate. These authors observed that products considered as bioequivalents presented in vitro dissolution differences, showing that in some cases in vitro dissolution can be more discriminating than in vivo bioequivalence testing. 

Population simulation results for products A1 and A2, B1 and B2, and C1 and C2 are presented in Table 6. 

The 90% CI ln-transformed of the ratio of *C*_max_ and AUC_0–t_ for the products and reference are within the 80–125% interval (Table 6), in accordance with the regulatory guidance of the FDA [32] and ANVISA [33] for bioequivalence evaluation of test and reference products. The average AUC_0–t_/AUC_0–inf_ ratios for the reference and all products were between 0.86 and 0.90, showing that the end time (96 h) used in the simulations was appropriate (>80%) to provide a reliable estimate of the extent of absorption [32,42]. 

## 4. Conclusions

Plasma concentration profile predictions and population simulation using virtual subjects obtained for fluconazole capsules with dissolution profiles that meet (A1, A2, B1, and B2) and do not meet (C1 and C2) the regulatory criterion of rapid or very rapid dissolution showed their bioequivalence. Computer simulations can be used as a tool for screening formulations that could be bioequivalent, contributing to gaining time and reducing costs for pharmaceutical companies.

## Figures and Tables

**Table 1:** Input data used in GastroPlus^™^ to simulate plasma concentrations.

Parameter	Value	Reference/Data Source
Solubility (mg/mL)	8.03 at pH 0.8;	[11,12]
	6.91 at pH 4.5;	
	7.82 at pH 6.8;	
	6.90 at pH 7.4	
pKa	2.56; 2.94; 11.01	[26,27]
Log P	0.82	ADMET Predictor^™^
Dose (mg)	150	[28,29]
Effective permeability, Peff (cm/s × 10^−4^)	4.06	ADMET Predictor^™^
Blood/plasma ratio	1.1	ADMET Predictor^™^
Unbound plasma (%)	27.41	ADMET Predictor^™^
Physiology	Human, fasting conditions	[28]
Body weight (kg)	61	[28]

**Table 2:** Elimination half-life (T1/2), coefficient of determination (R^2^), and Akaike Information Criterion (AIC) for compartmental models calculated by PKPlus^™^ module.

Compartmental Models	T1/2 (h)	R^2^	AIC
One-compartmental	29.55	0.9936	−80.27
Two-compartmental	30.25	0.9977	−87.53
Three-compartmental	1523.50	0.9976	−83.84

**Table 3:** Pharmacokinetic (PK) parameters from the two-compartmental model calculated by the PKPlus^™^ module.

Parameter	Value
Clearance, CL (L/h)	0.99565
Central compartment volume, Vc (L)	30.66
Elimination half-life, T1/2 (h)	30.25
Distribution rate constant from C1 to C2, K12 (h^−1^)	0.16515
Distribution rate constant from C2 to C1, K21 (h^−1^)	0.41884
Distribution volume of second compartment, V2 (L/kg)	0.19817

**Table 4:** Statistical parameters generated by GastroPlus^™^ for each predicted plasma concentration-time curve.

Product	*R* ^2^	SSE	RMSE	MAE
A1	0.987	1.482 × 10^−1^	1.161 × 10^−1^	7.561 × 10^−2^
A2	0.936	9.806 × 10^−1^	2.986 × 10^−1^	1.833 × 10^−1^
B1	0.929	1.097	3.158 × 10^−1^	1.898 × 10^−1^
B2	0.929	1.094	3.154 × 10^−1^	1.897 × 10^−1^
C1	0.832	2.382	4.653 × 10^−1^	2.402 × 10^−1^
C2	0.969	4.301 × 10^−1^	1.977 × 10^−1^	1.366 × 10^−1^

*R*^2^, coefficient of determination; SSE, sum of square error; RMSE, root mean square error; MAE, mean absolute error.

**Table 5:** Pharmacokinetic parameters of the reference product (Ref) based on the experimental curve and of products A1, A2, B1, B2, C1, and C2 obtained using simulated curves given by GastroPlus^™^.

Products	F%	*T*_max_ (h)	*C*_max_ (µg/mL)		AUC_0–t_ (µg h/mL)	
			Obs	Pred	Obs	Pred
Ref	90.0	4.00	3.49	--	135.77	--
A1	99.6	2.30	--	3.26	--	133.24
A2	99.6	1.66	--	3.82	--	132.48
B1	99.6	1.60	--	3.82	--	132.51
B2	99.6	1.60	--	3.82	--	132.51
C1	99.4	3.20	--	3.16	--	132.66
C2	99.6	1.80	--	3.81	--	132.37

%F, oral bioavailability: % of the drug that reached the systemic circulation; *T*_max_, time of *C*_max_; *C*_max_, maximum plasma concentration; AUC, area under the plasma concentration-time curve; Obs, observed value.

**Table 6:** Population simulation results: average pharmacokinetic (PK) parameters F%, *C*_max_, *T*_max_, AUC_0–inf_, AUC_0–t_, 90% CI ln-transformed for their ratio for A1, A2, B1, B2, C1, C2, and the reference product (Ref), and average AUC_0–t_/AUC_0–inf_ ratio.

PK Parameter	Ref	A1	A2	B1	B2	C1	C2
F%	90	99.62 (99.58–99.66)	99.53 (99.44–99.63)	99.55 (99.48–99.63)	99.58 (99.54–99.62)	99.25 (99.11–99.38)	99.64 (99.60–99.68)
*C*_max_ (µg/mL)	3.49	3.22 (3.06–3.40)	3.79 (3.61–3.98)	3.88 (3.71–4.07)	3.80 (3.65–3.96)	3.16 (3.02–3.31)	3.73 (3.58–3.89)
*T*_max_ (h)	4	2.24 (2.14–2.35)	1.66 (1.59–1.74)	1.60 (1.53–1.68)	1.60 (1.55–1.65)	3.36 (3.20–3.53)	1.77 (1.73–1.82)
AUC_0–inf_ (µg h/mL)	153.56	135.06 (126.60–144.10)	146.81 (135.90–158.60)	156.58 (146.50–167.30)	157.02 (148.30–166.30)	141.3 (129.3–154.4)	150.58 (142.10–159.60)
AUC_0–t_ (µg h/mL)	135.77	121.59 (115.70–127.80)	128.43 (121.50–135.70)	135.81 (129.30–142.60)	135.81 (129.30–142.70)	125.48 (117.20–134.40)	130.6 (124.90–136.60)
Average AUC_0–t_/AUC_0–inf_	0.88	0.90	0.87	0.87	0.86	0.88	0.87
